# Supplementary material for: Binding pose depth modulates photoswitchable ligands’ efficacy at the 5-HT2A receptor
Source: Commun Chem. 2026 Feb 12;9:121. doi: 10.1038/s42004-026-01936-5 (PMC12992696; doi:10.1038/s42004-026-01936-5)
Supplement: Supplementary file 2 — Supplementary Information [file 42004_2026_1936_MOESM2_ESM.pdf]

Supplementary Information:

Binding pose depth modulates photoswitchable ligands' efficacy at the 5-HT_{2A} receptor

Verena Weber,[1,2,†] Giacomo Salvadori,[2,†,*] Federico Natale,[1,2] Hubert Gerwe,[3] Michael Decker,[3] Paolo Carloni,[2,*] and Giulia Rossetti[2,4,5,*]

[1] RWTH Aachen University, 52056 Aachen, Germany

[2] Computational Biomedicine, Institute for Neuroscience and Medicine INM-9, Forschungszentrum Jülich GmbH, 52428 Jülich, Germany

[3] Julius-Maximilians-Universität Würzburg (JMU), Institut für Pharmazie und Lebensmittelchemie, Pharmazeutische und Medizinische Chemie, 97074 Würzburg, Germany;

[4] Department of Neurology, Medical Faculty, RWTH Aachen University, 52074 Aachen, Germany

[5] Jülich Supercomputing Center (JSC), Forschungszentrum Jülich, 52425 Jülich, Germany

[†] These authors contributed equally

[*] Email: g.salvadori@fz-juelich.de, p.carloni@fz-juelich.de, g.rossetti@fz-juelich.de

Supplementary Figures

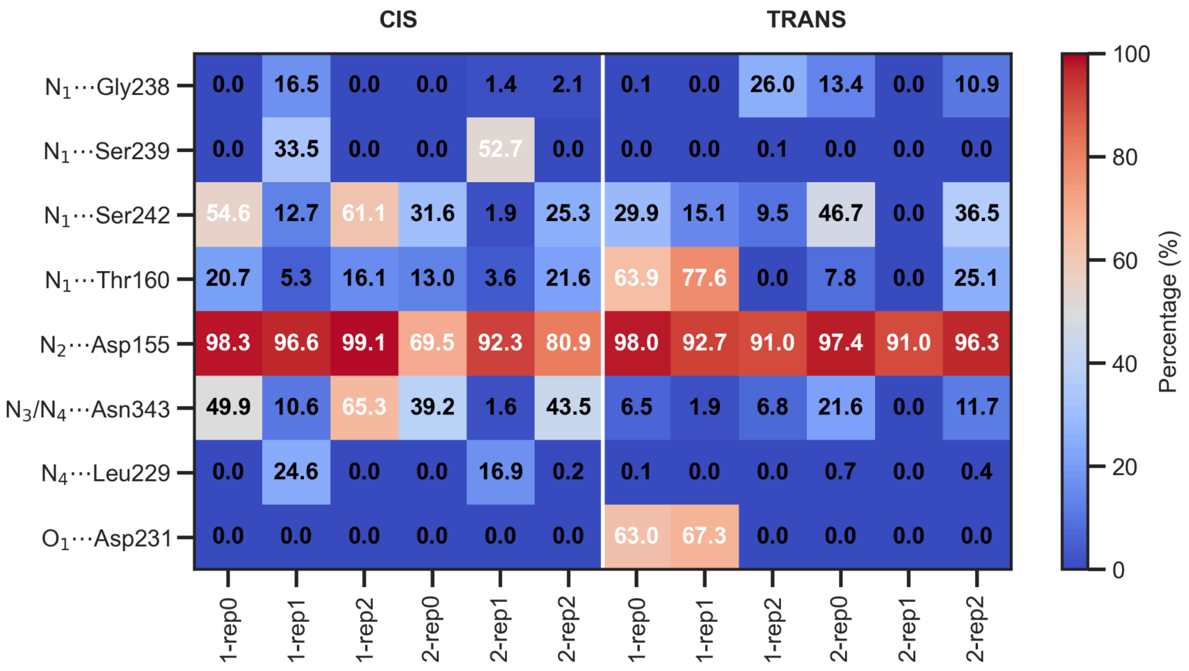

Figure S1: Hydrogen-bond interaction analysis in the inactive 5-HT$_{2A}$ receptor. Heatmap showing hydrogen bond occupancy (distance cutoff 3.2Å, angle cutoff 125°) between ligand heteroatoms and receptor residues across all MD trajectories. Data represents three independent replicas for compounds **1** and **2** in both *cis* (left) and *trans* (right) configurations bound to the inactive receptor.

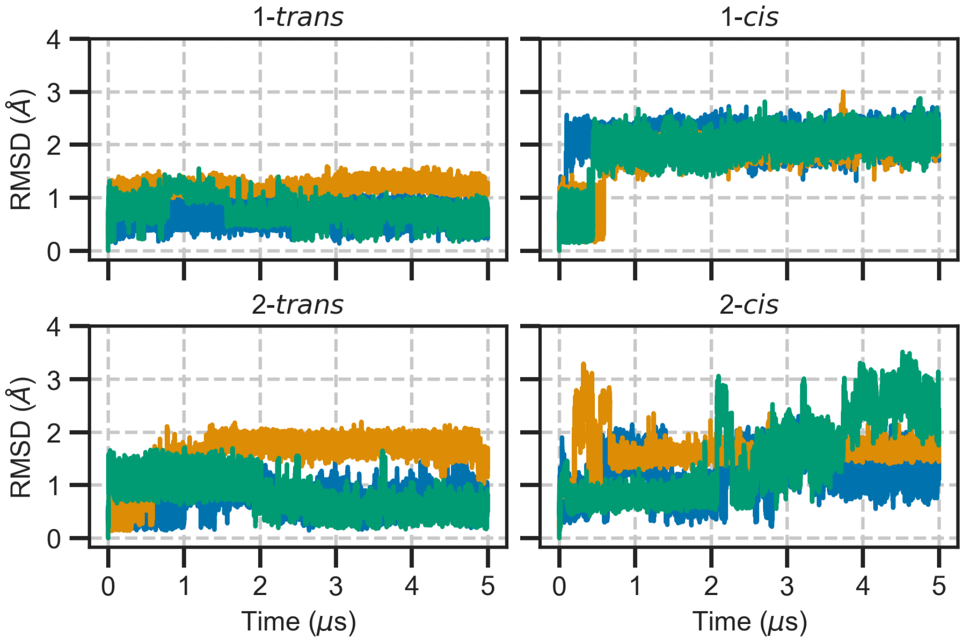

Figure S2: Ligand conformational stability in inactive 5-HT$_{2A}$ receptor. Heavy-atom RMSD trajectories of compounds **1** and **2** (*cis/trans* isomers) relative to the first frame of the production run over 5 μs simulations. Three independent replicas shown in different colors.

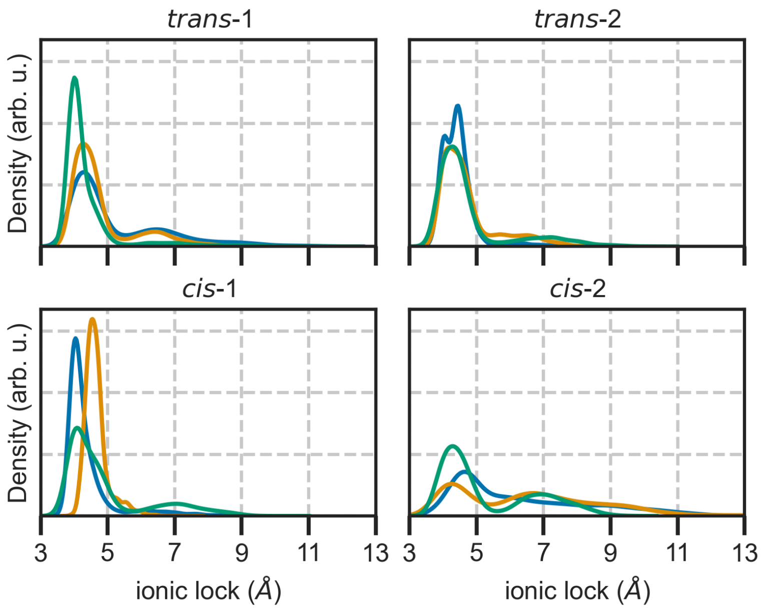

Figure S3: **Kernel-density distributions (KDE) of the Arg173$^{3.50}$–Glu318$^{6.30}$ ionic–lock distance in the inactive 5-HT$_{2A}$ receptor.** Each panel shows the KDE for one ligand isomer; blue, orange, and green lines correspond to the three replicas.

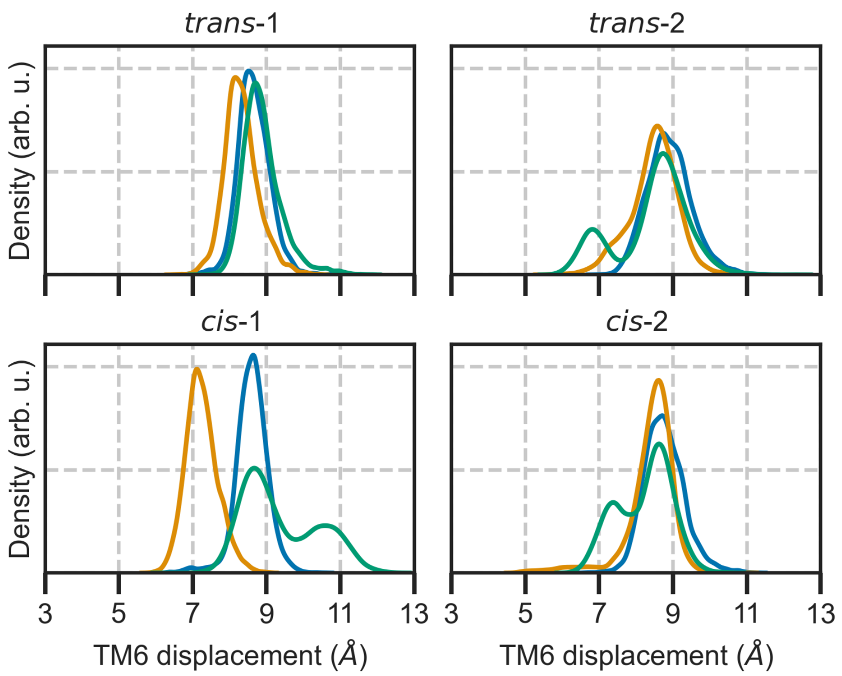

Figure S4: Kernel-density distributions of the TM3-TM6 cytoplasmic distance in the inactive 5-HT$_{2A}$ receptor. Each panel shows the KDE for one ligand isomer; blue, orange, and green lines correspond to the three replicas.

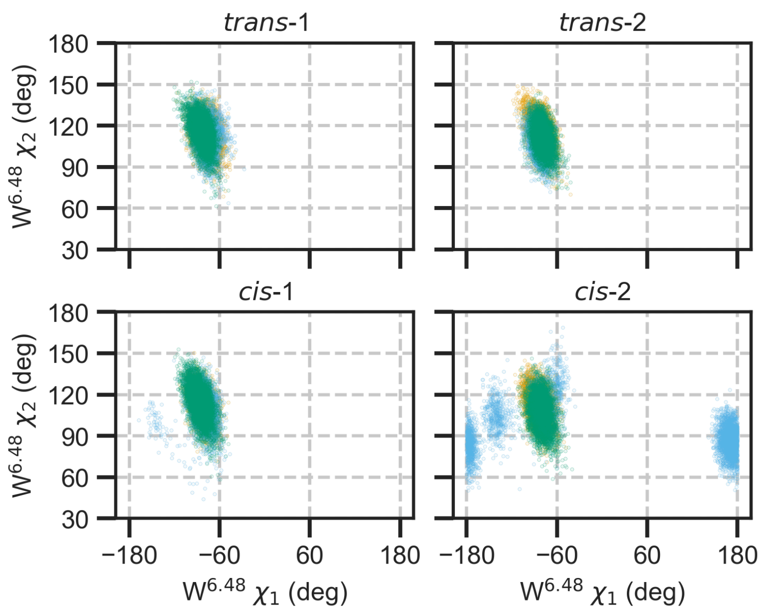

Figure S5: **Toggle switch conformational analysis in the inactive 5-HT$_{2A}$ receptor.** Correlation plots of Trp336$^{6.48}$ χ1 vs χ2 dihedral angles; blue, orange, and green lines correspond to the three replicas.

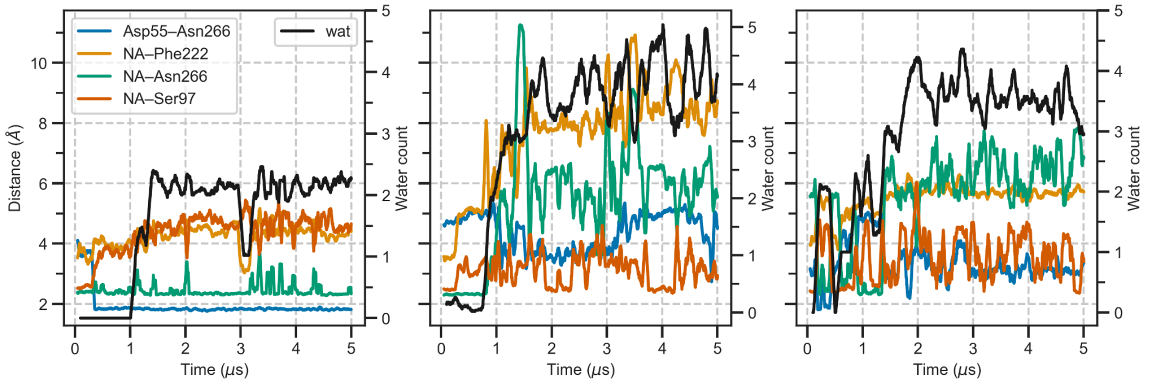

Figure S6: Na$^+$ coordination and hydration in the inactive *trans*-1 system. Plots show distances to key coordinating residues (colored lines) and water count within Na$^+$ coordination sphere (black line, secondary axis). Each box is a different replica.

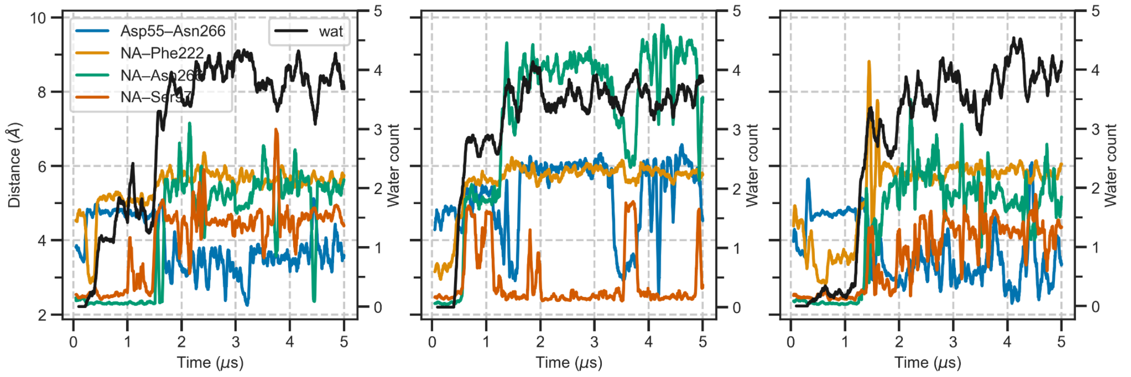

Figure S7: Na$^+$ coordination and hydration in the inactive *trans*-2 system. Plots show distances to key coordinating residues (colored lines) and water count within Na$^+$ coordination sphere (black line, secondary axis). Each box is a different replica.

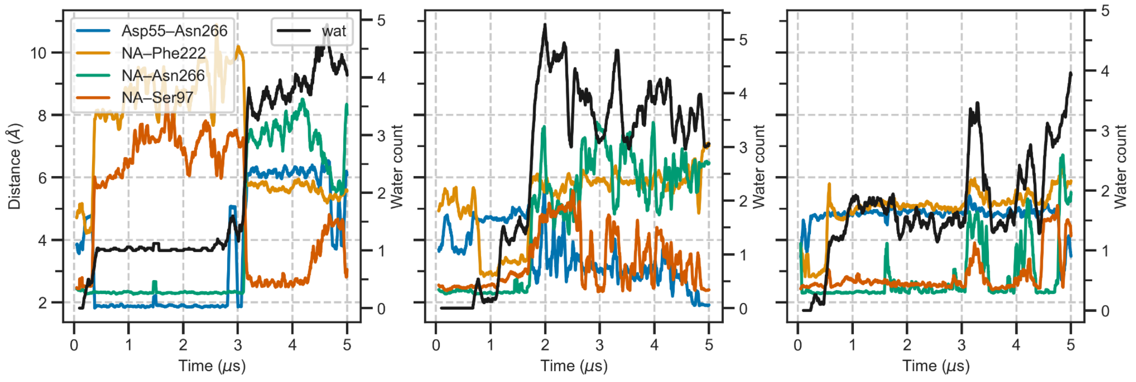

Figure S8: Na⁺ coordination and hydration in the inactive *cis*-1 system. Plots show distances to key coordinating residues (colored lines) and water count within Na⁺ coordination sphere (black line, secondary axis). Each box is a different replica.

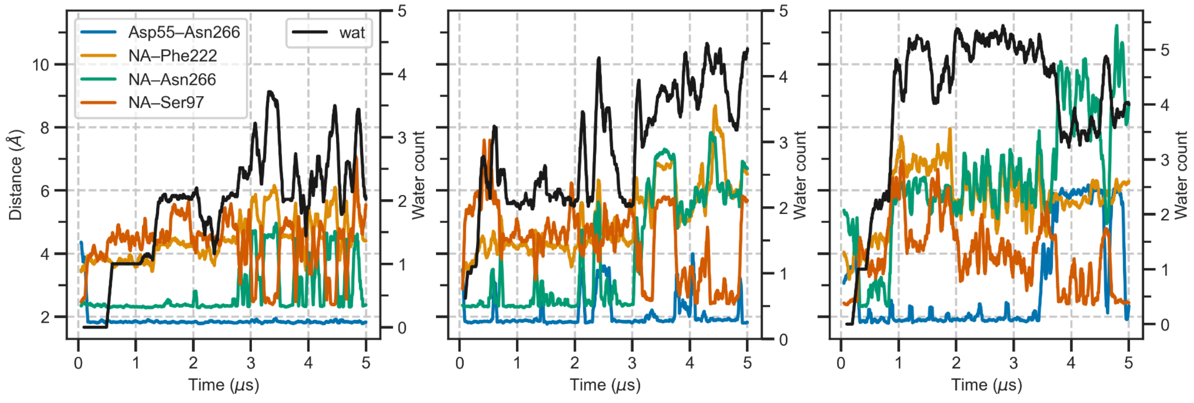

Figure S9: Na⁺ coordination and hydration in the inactive *cis*-2 system. Plots show distances to key coordinating residues (colored lines) and water count within Na⁺ coordination sphere (black line, secondary axis). Each box is a different replica.

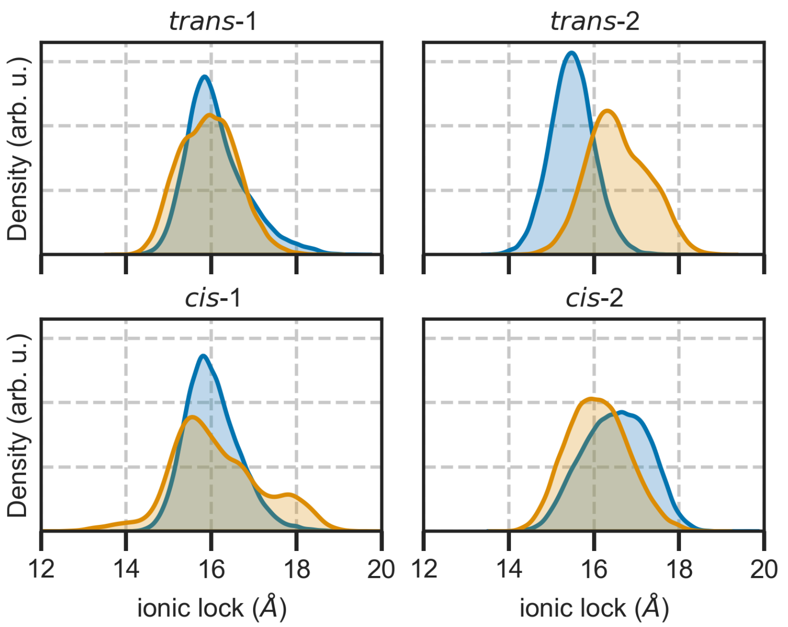

Figure S10: Kernel-density distributions of the Arg173$^{3.50}$–Glu318$^{6.30}$ ionic–lock distance in the active 5-HT$_{2A}$ receptor. Each panel shows the KDE for one ligand isomer; blue and orange lines correspond to the two replicas.

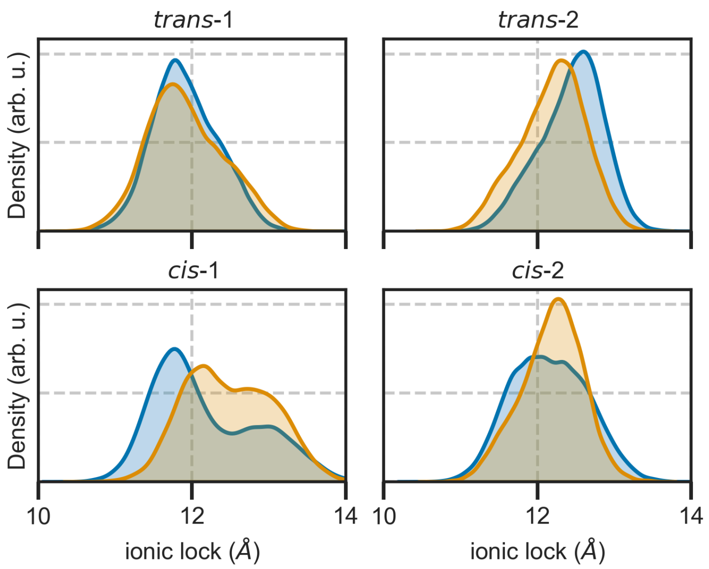

Figure S11: Kernel-density distributions of the TM3-TM6 cytoplasmic distance in the active 5-HT$_{2A}$ receptor. Each panel shows the KDE for one ligand isomer; blue and orange lines correspond to the two replicas.

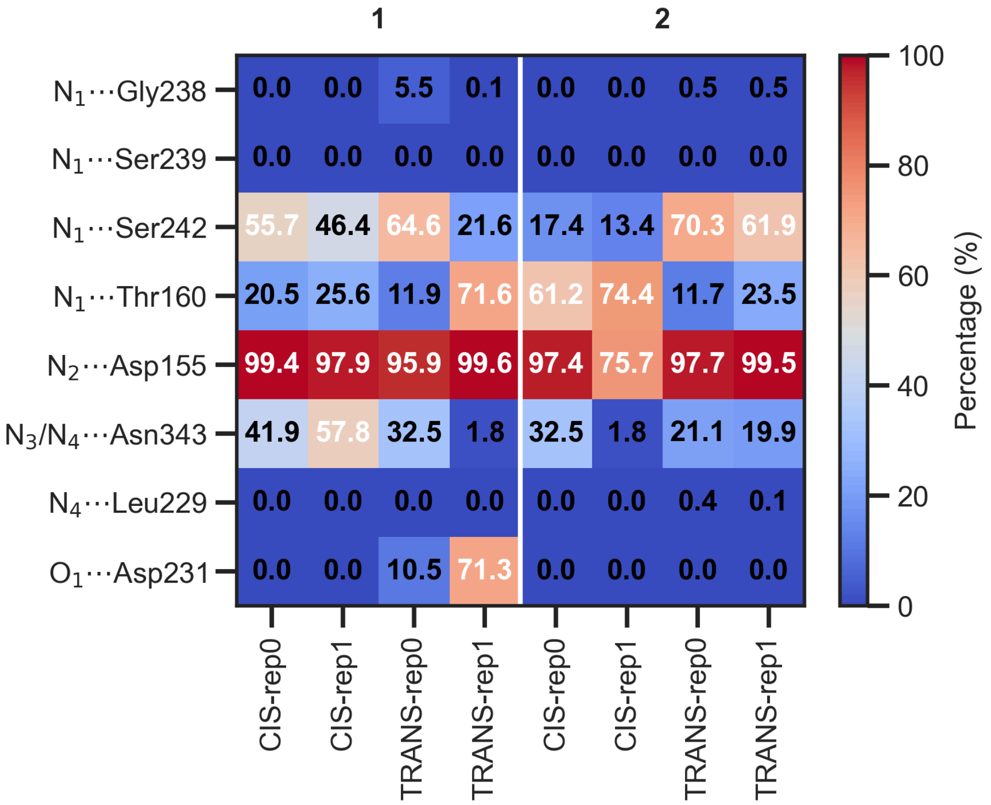

Figure S12: Hydrogen-bond interaction analysis in the active 5-HT$_{2A}$ receptor. Heatmap showing hydrogen bond occupancy (distance cutoff 3.2Å, angle cutoff 125°) between ligand heteroatoms and receptor residues across all MD trajectories. Data represents two independent replicas for compounds **1** and **2** in both *cis* (left) and *trans* (right) configurations bound to the active receptor.

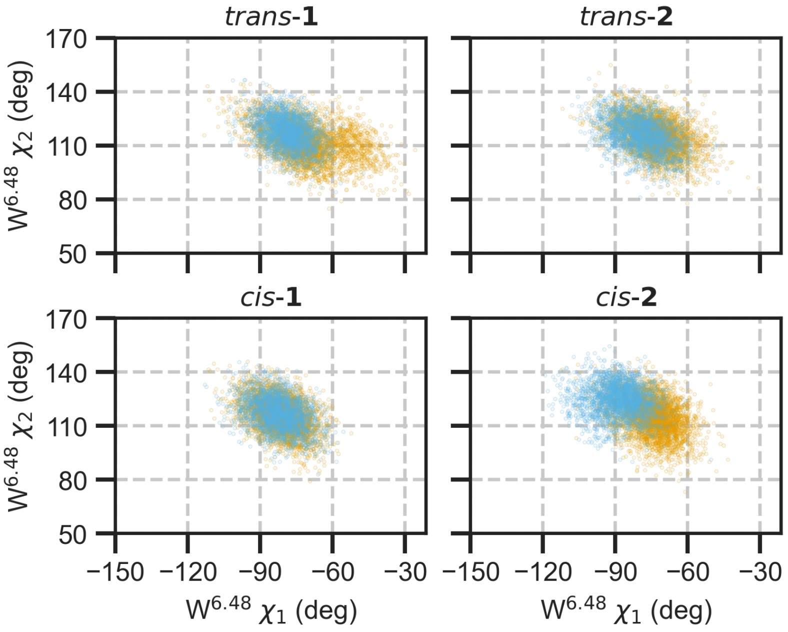

Figure S13: Toggle switch conformational analysis in the active 5-HT$_{2A}$ receptor. Correlation plots of Trp336$^{6.48}$ χ1 vs χ2 dihedral angles; blue and orange dots correspond to the two replicas.

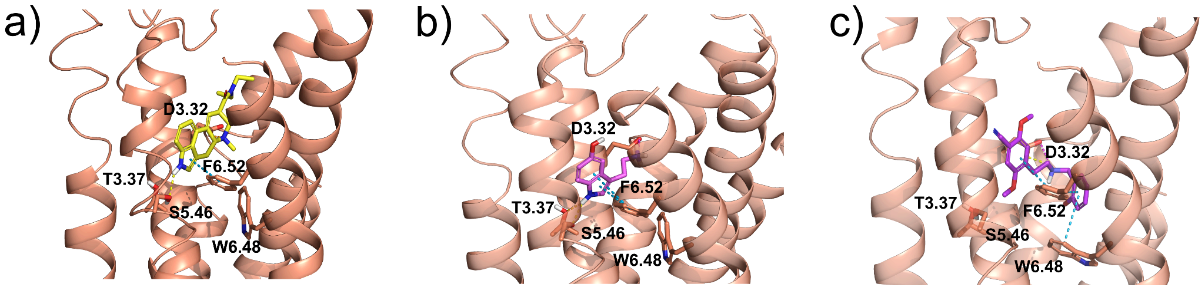

Figure S14: Binding poses of 5-HT$_{2A}$ agonists in the orthosteric binding pocket. a) The partial agonist LSD forms interactions with Asp155$^{3.32}$, Ser242$^{5.46}$ and Phe340$^{6.52}$. b) The endogenous ligand serotonin interacts with Asp155$^{3.32}$, Ser242$^{5.46}$ and Phe340$^{6.52}$. c) The 5-HT$_{2A}$ selective agonist 25CN-NBOH exhibits an interaction profile involving Asp155$^{3.32}$, Ser242$^{5.46}$, Phe340$^{6.52}$, Trp336$^{6.48}$. The active receptor structure is represented as a salmon cartoon, while the agonist compounds are displayed in yellow, pink and magenta licorice representations, respectively. Salt bridges are represented as magenta dotted lines, hydrogen bonds are represented as yellow dotted lines and π-π stacking interactions are represented as cyan dotted lines.

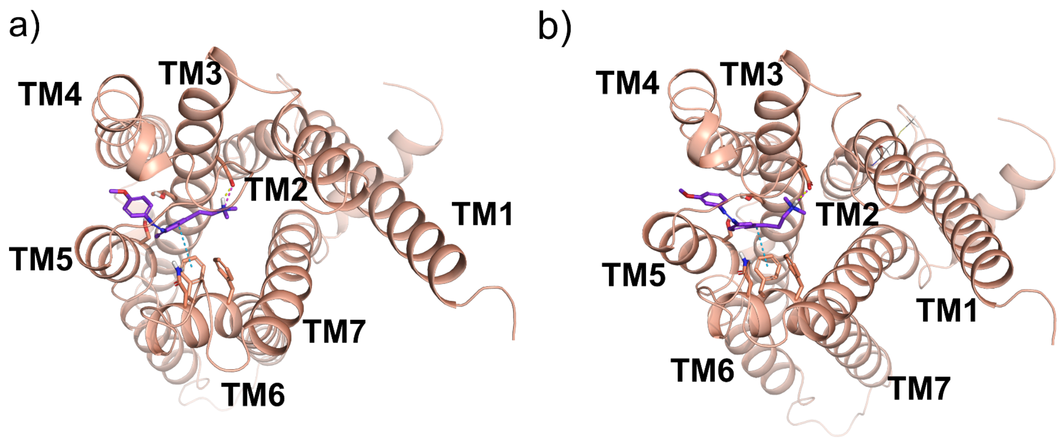

Figure S15: The hydrophobic tunnel formed in between TM4 and TM5. a) The active receptor state in complex with *cis*-**1** sterically clashes with TM4. b) The active receptor state in complex with *cis*-**2** shows that the m-methoxy phenyl moiety wedges in the hydrophobic tunnel. The active receptor structure is represented as a salmon cartoon, while the ligands are displayed in a violet licorice representation, respectively. Salt bridges are represented as magenta dotted lines, hydrogen bonds are represented as yellow dotted lines and π-π stacking interactions are represented as cyan dotted lines.

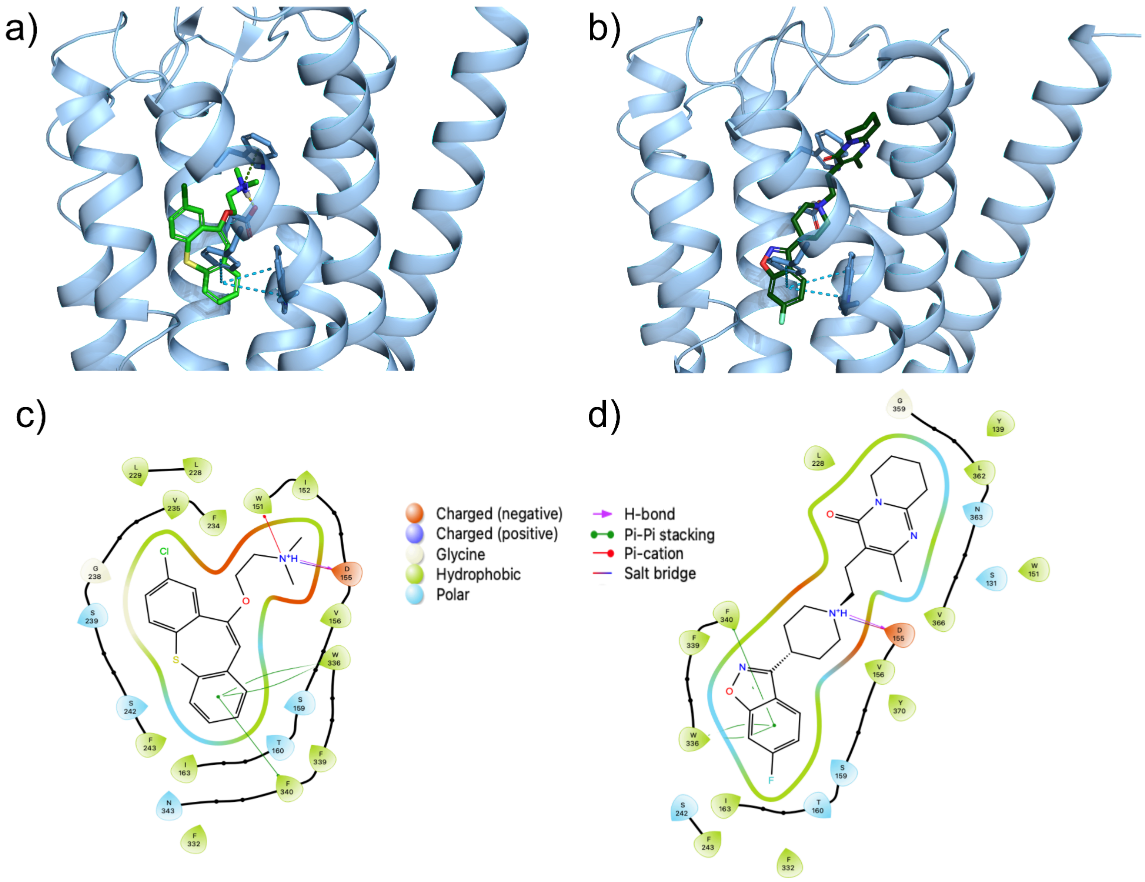

Figure S16: 5-HT$_{2A}$ antagonists engage with residues located at the bottom of the orthosteric binding pocket. A) The interaction profiles of zotepine (a) and risperidone (b) include Asp155$^{3.32}$, Phe340$^{6.52}$ and Trp336$^{6.48}$ facilitate the deep insertion of both antagonists in the binding pocket and stabilize the vertical orientation of the toggle switch. The inactive receptor structure is represented as a blue cartoon, while the ligands are displayed in green licorice representations, respectively. Salt bridges are represented as magenta dotted lines, hydrogen bonds are represented as yellow dotted lines, cation-π interactions are represented as dark green dotted lines and π-π stacking interactions are represented as cyan dotted lines. Despite their unique chemical scaffolds, the protein-ligand interaction diagram of zotepine (c) and risperidone (d) show a high level of similarity.

Supplementary tables

Table S1 Molecular dynamics simulation table for photoswitchable 5-HT$_{2A}$ ligands. Eight independent systems were built to evaluate both isomeric forms of compounds **1** and **2** in inactive and G$_q$-coupled active 5-HT$_{2A}$ receptor states. For each condition we report: receptor conformational template ("Inactive" from PDB 6A94; "Active" from PDB 6WHA), ligand isomer (*trans* or *cis*), inclusion of the G$_q$ subunit, the number of statistically independent replicas, and the simulation time per replica (in microseconds).

#MD	Receptor	Ligand	G$_q$ subunit	Replica count	Time (µs)
1	Inactive	**1**-*trans*	No	3	5
2	Inactive	**1**-*cis*	No	3	5
3	Active	**1**-*trans*	Yes	2	3.76<br>2.46
4	Active	**1**-*cis*	Yes	2	3.66<br>2.58
5	Inactive	**2**-*trans*	No	3	5
6	Inactive	**2**-*cis*	No	3	5
7	Active	**2**-*trans*	Yes	2	3.60<br>2.38
8	Active	**2**-*cis*	Yes	2	3.96<br>2.62

Table S2 Microswitch metrics For each ligand isomer (**1**-*trans*, **1**-*cis*, **2**-*trans*, **2**-*cis*) we report the average value and the standard deviation (between parenthesis) for each microswitch obtained from three independent microsecond MD replicas in the inactive receptor and from two replicas in the active (Gq-coupled) receptor. The ALL rows give the respective values considering all the replicas for each condition. For χ_1 and χ_2, we report the circular mean and the circular standard deviation. "N" represents the number of frames.

**Receptor state**	**Ligand isomer**	**Replica**	**N**	**d_{TM} (Å)**	**d_{IL} (Å)**	**$W^{6.48}$ χ_1 (deg)**	**$W^{6.48}$ χ_2 (deg)**
**Inactive**	**1**-*trans*	0	5000	8.7 (0.4)	5.3 (1.5)	–77.6 (9.7)	113.5 (10.0)
		1	5000	8.3 (0.5)	4.8 (1.0)	–77.2 (9.5)	112.0 (10.6)
		2	5000	8.9 (0.5)	4.4 (0.9)	-83.2 (9.2)	115.4 (10.4)
		ALL	15000	8.6 (0.5)	4.8 (1.2)	-79.4 (9.9)	113.6 (10.4)
	**2**-*trans*	0	5000	8.9 (0.6)	4.4 (0.6)	-81.6 (9.0)	115.5 (9.9)
		1	5000	8.5 (0.7)	4.7 (0.9)	-83.4 (7.5)	108.1 (9.3)
		2	5000	8.4 (1.0)	4.8 (1.2)	-79.3 (8.2)	112.2 (9.6)
		ALL	15000	8.6 (0.8)	4.7 (0.9)	-81.4 (8.4)	111.9 (10.1)
	**1**-*cis*	0	5000	8.6 (0.4)	4.4 (0.7)	-83.1 (8.2)	111.3 (9.2)
		1	5000	7.2 (0.4)	4.7 (0.4)	-81.7 (11.3)	110.9 (11.1)
		2	5000	9.4 (1.0)	4.9 (1.3)	-86.8 (10.0)	115.1 (11.9)
		ALL	15000	8.6	4.7	-83.9	112.4

				(0.8)	(0.9)	(10.1)	(10.9)
	**2**-*cis*	0	5000	8.8<br>(0.5)	6.4<br>(2.0)	-84.6<br>(9.3)	111.7<br>(10.2)
		1	5000	8.4<br>(0.7)	6.6<br>(2.3)	-157.8<br>(51.8)	95.3<br>(17.0)
		2	5000	8.2<br>(0.7)	5.3<br>(1.4)	-81.6<br>(9.1)	106.9<br>(11.3)
		ALL	15000	8.5<br>(0.7)	6.1<br>(2.0)	-95.1<br>(38.6)	106.2<br>(14.2)
**Active**	**1**-*trans*	0	3760	11.9<br>(0.4)	16.1<br>(0.7)	-70.6<br>(12.8)	112.4<br>(9.8)
		1	2460	11.9<br>(0.5)	15.9<br>(0.7)	-78.8<br>(7.5)	117.5<br>(8.6)
		ALL	6220	11.9<br>(0.5)	16.0<br>(0.7)	-73.9<br>(11.7)	114.4<br>(9.7)
	**2**-*trans*	0	3600	12.4<br>(0.4)	15.5<br>(0.5)	-73.6<br>(9.3)	115.3<br>(9.4)
		1	2380	12.2<br>(0.4)	16.6<br>(0.7)	-79.1<br>(9.2)	116.1<br>(9.2)
		ALL	5980	12.3<br>(0.4)	15.9<br>(0.8)	-75.8<br>(9.6)	115.6<br>(9.4)
	**1**-*cis*	0	3660	12.2<br>(0.7)	16.0<br>(0.6)	-81.6<br>(8.2)	115.9<br>(9.3)
		1	2580	12.5<br>(0.6)	16.1<br>(1.1)	-82.5<br>(8.2)	117.0<br>(8.9)
		ALL	6240	12.3<br>(0.7)	16.1<br>(0.9)	-82.0<br>(8.2)	116.4<br>(9.1)
	**2**-*cis*	0	3960	12.2<br>(0.5)	16.5<br>(0.8)	-74.9<br>(8.7)	115.5<br>(10.1)
		1	2380	12.2<br>(0.4)	16.1<br>(0.7)	-88.4<br>(8.9)	124.5<br>(8.7)
		ALL	6340	12.2<br>(0.5)	16.3<br>(0.8)	-80.5<br>(11.0)	119.2<br>(10.6)

Supplementary Note 1: Molecular docking

Initial binding poses for the *cis* and *trans* isomers of both compound **1** and compound **2** within the active (PDB ID: 6WHA) and inactive (PDB ID: 6A94) receptor structures were generated using molecular docking. Schrödinger's Glide (version 2022-1) was used with its standard precision (SP) docking procedure. For the active receptor structure, the docking grid center was defined based on the binding position of LSD in the 5-HT_{2A}R-LSD-G_i structure (PDB ID: 6WGT). The indole ring of LSD was used as a core constraint with an RMSD tolerance of 2.0 Å to guide pose selection. A H-bond constraint was applied to ensure interaction with the canonical $Asp155^{3.32}$. For the inactive receptor structure, a similar approach was used, with the grid center positioned to encompass the orthosteric binding site occupied by methiothepin. In all docking runs, the top-scoring poses were selected based on GlideScore and visual inspection, ensuring the characteristic salt bridge to $Asp155^{3.32}$ was formed and the indole moiety maintained an expected orientation within the binding pocket. Representative docking poses are shown in Fig. S18 and Fig. S19. In all complexes, we observed a conserved salt bridge between the protonated tertiary amine of the respective ligand and $Asp155^{3.32}$ (Fig. S18). This ionic interaction serves as the primary anchoring point in the orthosteric pocket, similar to the binding mode observed for endogenous serotonin and other tryptamine derivatives. Moreover, the indole moiety established π-π stacking interactions with the aromatic cluster composed of $Phe339^{6.51}$ and $Phe340^{6.52}$ in all docking poses but cis-1 in the inactive receptor structure.

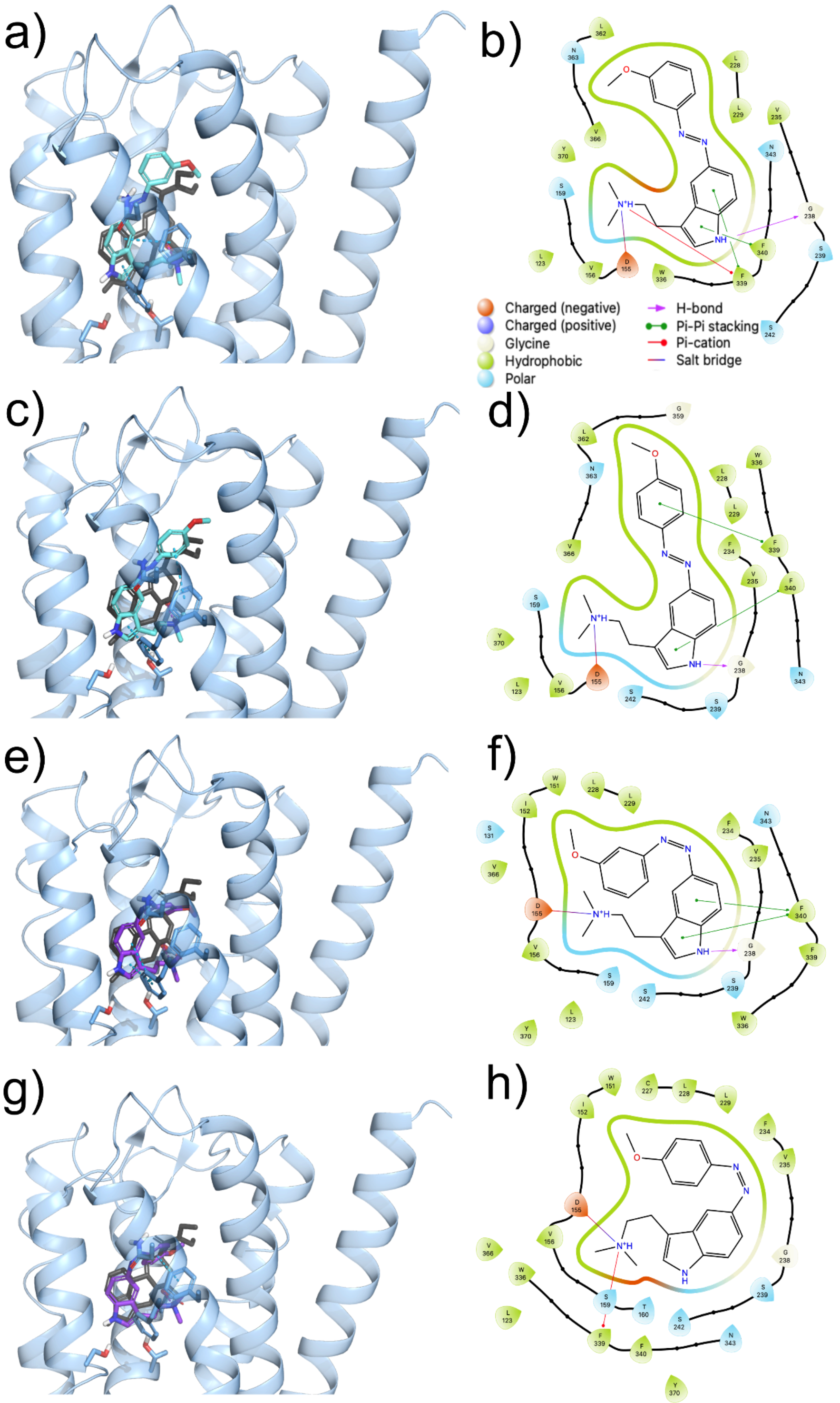

Figure S18: Docking poses of photoswitchable molecules in the inactive receptor structure. a-b) docking pose of trans-2, c-d) docking pose of trans-1, e-f) docking pose of cis-2, g-h) docking pose of cis-1. The inactive receptor structure is represented as a blue cartoon, trans conformers are displayed in a cyan licorice representation, cis conformers are displayed in a violet licorice representation and

the reference ligand LSD is displayed in a black licorice representation. Salt bridges are represented as magenta dotted lines, hydrogen bonds are represented as yellow dotted lines and π-π stacking interactions are represented as cyan dotted lines.

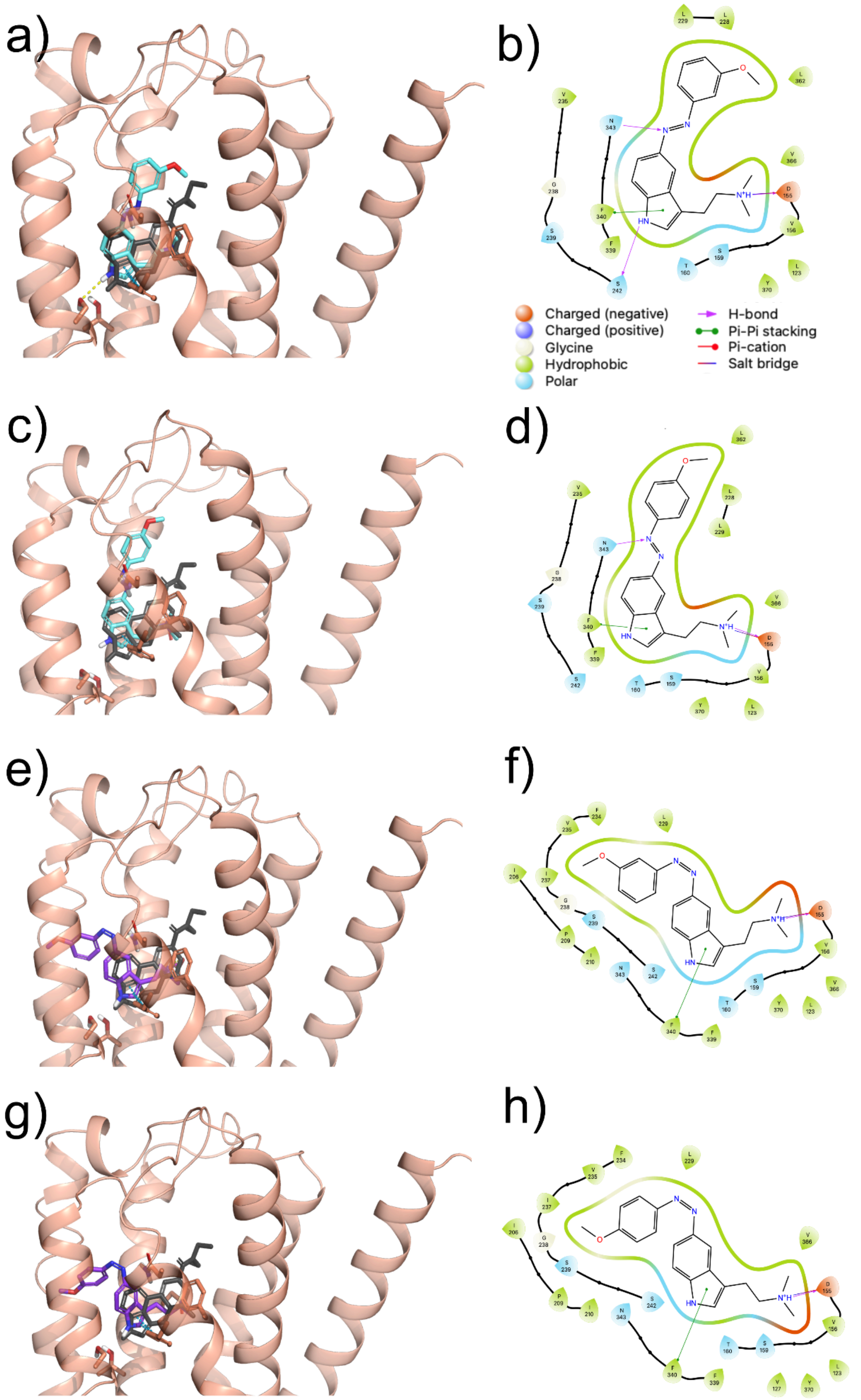

Figure S19: Docking poses of photoswitchable molecules overlaid on the reference molecule LSD in the active receptor structure. a-b) docking pose of trans-2, c-d) docking pose of trans-1, e-f) docking pose of cis-2, g-h) docking pose of cis-1. The active receptor structure is represented as a salmon cartoon, trans conformers are displayed in a cyan licorice representation, cis conformers are displayed in a violet licorice representation and the reference ligand LSD is displayed in a black licorice representation. Salt bridges are represented as magenta dotted lines, hydrogen bonds are represented as yellow dotted lines and π-π stacking interactions are represented as cyan dotted lines.

Supplementary Note 2: Secondary poses

In addition to the dominant binding modes described in the main text, our molecular dynamics simulations revealed several secondary binding poses and transient interactions that provide additional insight into the conformational dynamics of compounds **1** and **2**. For all secondary poses, the global activation metrics are unchanged.

Inactive receptor

***trans*-1** A secondary pose in which the Asp231$^{5.35}$ contact breaks allows *trans*-**1** to tilt toward TM5 (Fig. S20) and form transient H-bond interactions with Ser239$^{5.44}$ (9%) and Gly238$^{5.43}$ (10%). Here, trans-1 partially mimics LSD, but it still misses the Ser242/Asn343 interactions. Notably, the solvation around the methoxy group increases significantly (Figure 2).

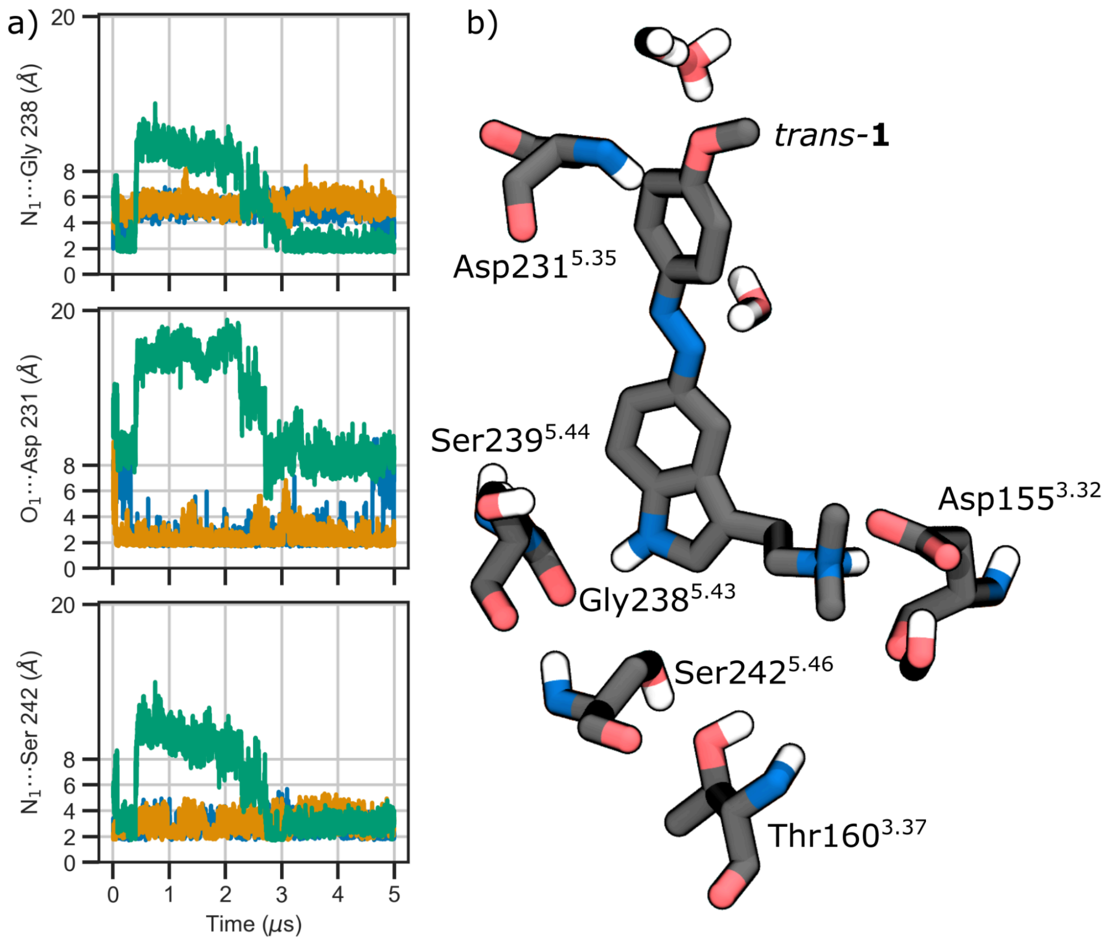

Figure S20: a) Hydrogen bonding distances between *trans*-**1** and Gly238, Asp231, and Ser242. b) Licorice representation of *trans*-**1** and the main close-by residues. The water molecules within 3.2A from ligand's oxygen and nitrogen atoms are shown.

trans-2 trans-2 assumes a vestibular pose (one replica out of three): The ligand shifts upward in the binding pocket, losing most polar contacts except the Asp155$^{3.32}$ salt bridge (that is however weakened) and forming interactions with water molecules (Figure S21).

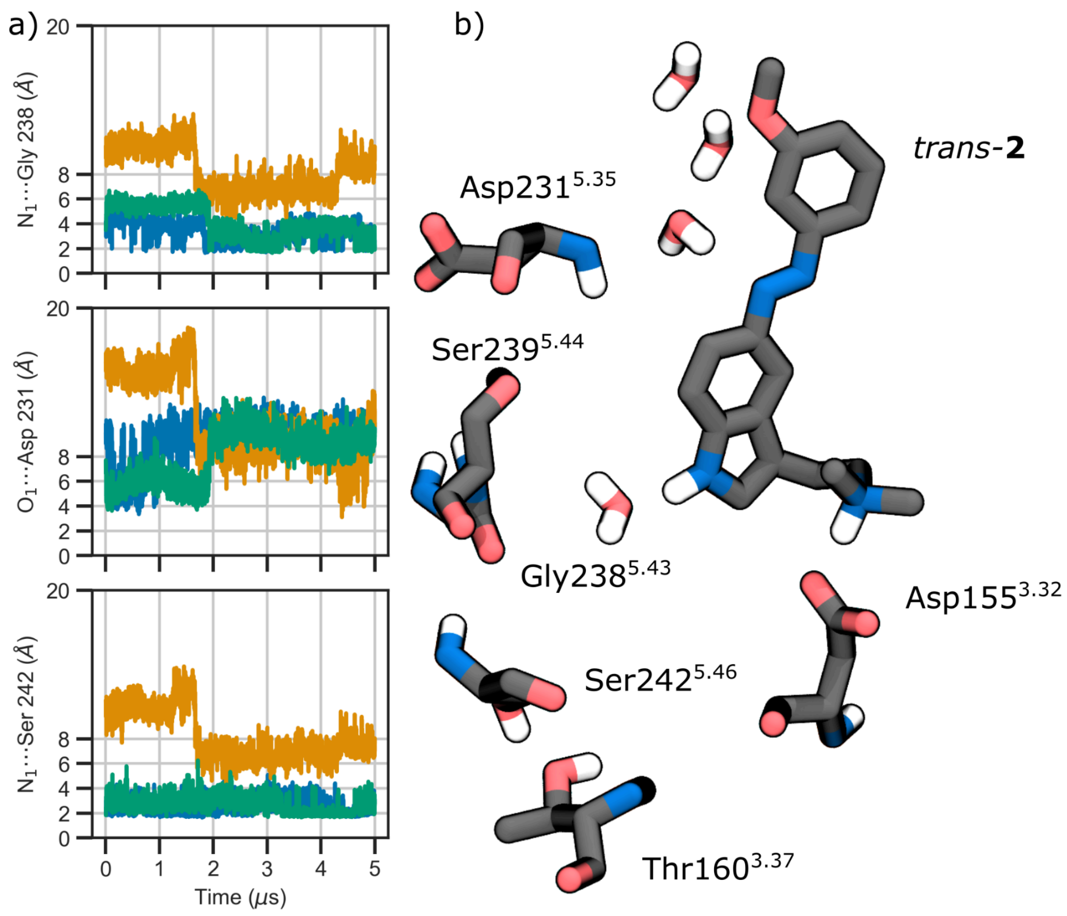

Figure S21: a) Hydrogen bonding distances between *trans*-**2** and Gly238, Asp231, and Ser242. b) Licorice representation of *trans*-**2** and the main close-by residues. The water molecules within 3.2A from ligand's oxygen and nitrogen atoms are shown.

***cis*-1** We identified a significant secondary pose triggered by rotation around the C-C-N=N (azobenzene) dihedral angle. This conformational change, observed in one out of three replicas, results in cleavage of the hydrogen bonds with Ser242$^{4.57}$ and Asn233$^{5.44}$. Consequently, the indole ring undergoes a reorientation, pointing directly toward either Ser239$^{4.54}$ or Gly238 (Figure S22).

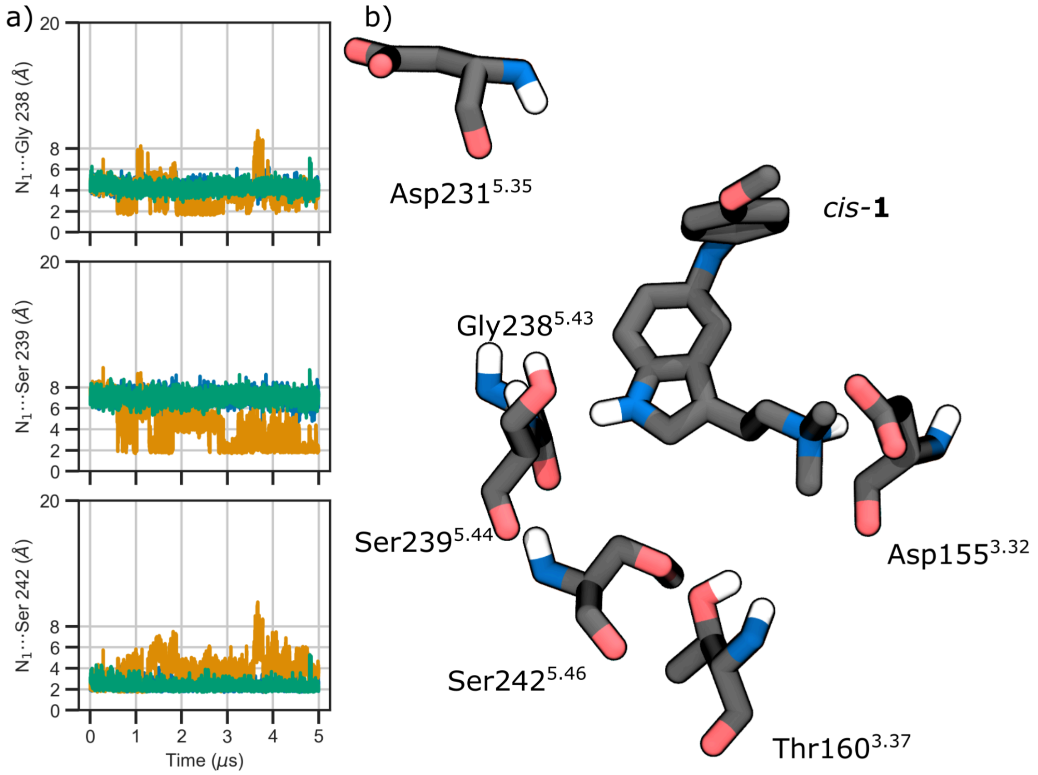

Figure S22: a) Hydrogen bonding distances between *trans*-**2** and Gly238, Asp231, and Ser242. b) Licorice representation of *trans*-**2** and the main close-by residues. The water molecules within 3.2A from ligand's oxygen and nitrogen atoms are shown.

This conformational change is also associated with the transient twisting of the toggle switch (Figure S23).

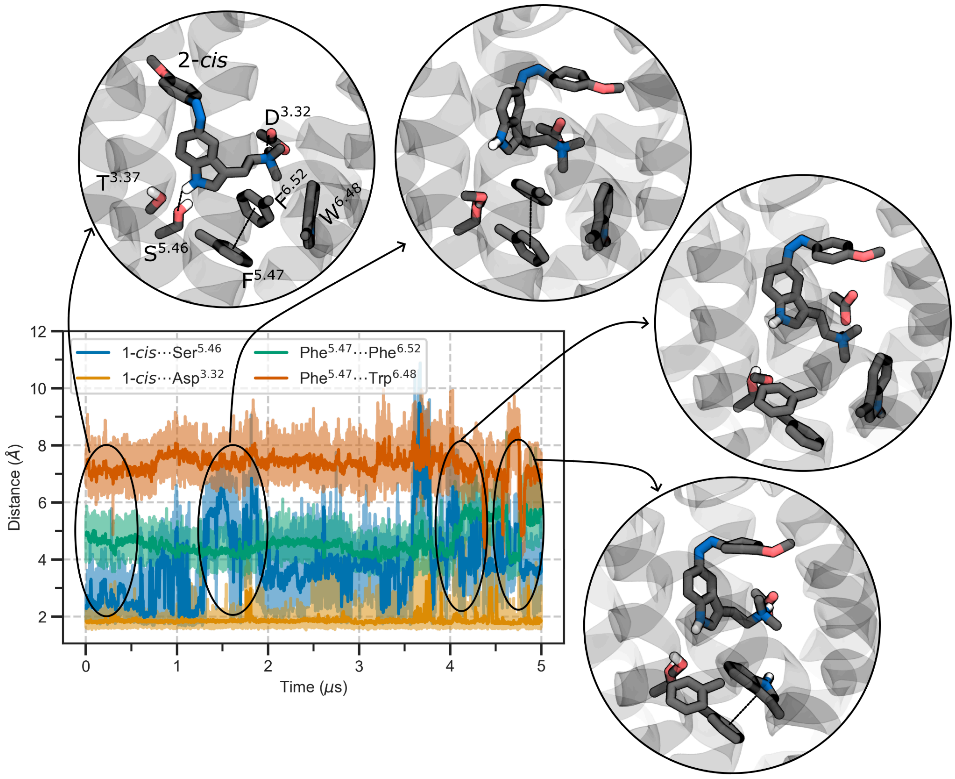

Figure S23: *Cis*-1 triggers toggle switch rotation in the inactive receptor. Moving average (window of 20 frames) of the time evolution of four intermolecular distances. Key aromatic or polar side chains are shown as sticks and labelled by Ballesteros-Weinstein index. Top panel, four representative snapshots extracted from the distance plot.

***cis*-2** The high mobility of ***cis*-2** results in a remarkable conformational event not observed with other ligands. At the end of two replicas out of three, it undergoes complete dissociation from the conserved Asp155$^{3.32}$ salt bridge (Figure S24), an interaction maintained by all other ligands throughout their simulations. This loss of the primary anchoring interaction allows ***cis*-2** to drift into the extracellular vestibular region of the receptor, adopting a loosely bound pose characterized by minimal protein contacts. In this vestibular state, the ligand interacts mainly with water molecules.

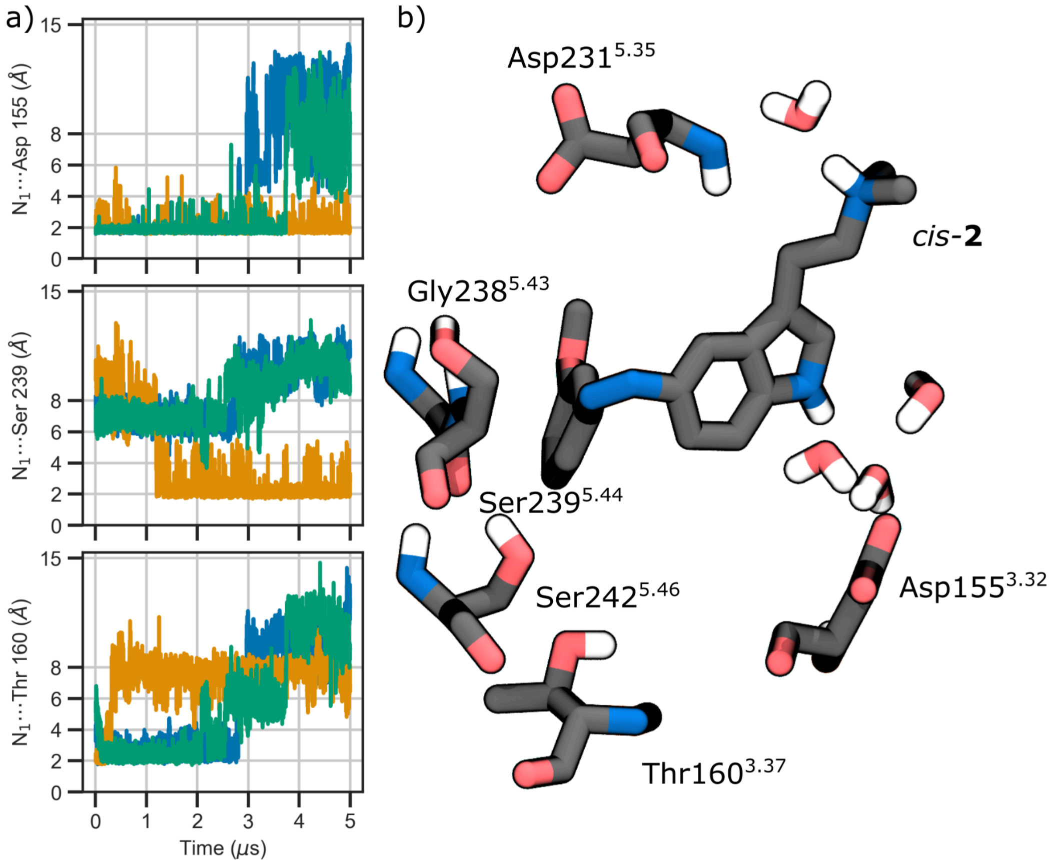

Figure S24: a) Hydrogen bonding distances between ***cis*-2** and Asp155, Ser239, and Thr160. b) Licorice representation of ***cis*-2** and the main close-by residues. The water molecules within 3.2A from ligand's oxygen and nitrogen atoms are shown.

Supplementary Note 3: Parametrization of the ligands

The molecular structures of compounds **1** and **2** are presented in Figure 1b. For each isomer, 30 representative conformations were extracted from a 30 ps-long QM/MM MD of the ligand solvated in a truncated octahedral TIP3P water box. The ligand was treated at the DFTB3 level of theory with a 10 Å cutoff for QM/MM interactions. All QM/MM MD simulations were performed with AMBER2020 at 300 K in the NPT ensemble. RESP charges were computed using multiconformational fitting (30 structures) with AMBER's PyRESP,[1] based on B3LYP-D3/6-31G(d,p) ESP

calculations on geometries optimized at the B3LYP-D3/6-31G(d) level. The ligands were parameterized using the GAFF force field (modified for the azobenzene[2]), with reparameterization of the C=C-N=N dihedrals to match our DFT reference (Figure S25). All QM calculations were performed with Gaussian09.

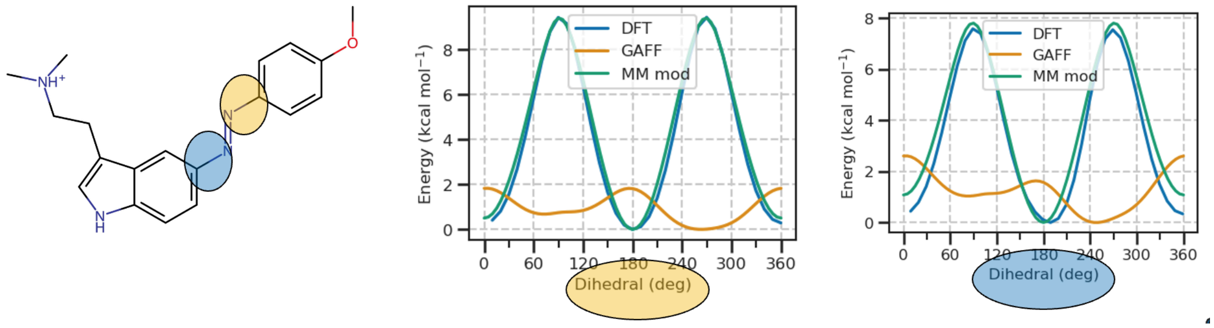

Figure S25: Forcefield parametrization Scan of the two C=C-N=N dihedrals performed with GAFF, DFT, and our modified forcefield.

Supplementary Note 4: Mutational analysis

To further validate our findings, we performed additional MD simulations of selected key receptor mutants. As a control, we simulated the D155A mutation, which experimentally abolishes tryptamine binding[3]. Within 1 μs, the ligand did not fully dissociate. This is consistent with the expectation that unbinding events typically occur on long timescales due to relatively high free energy barriers, much larger than kT. However, it underwent significant repositioning, consistent with the reported loss of affinity. Rather than anchoring through its protonated tail that now interacts with Cys227, the ligand shifts such that the indole NH group forms a water-mediated hydrogen bond with the Ala155 backbone (Figure S26).

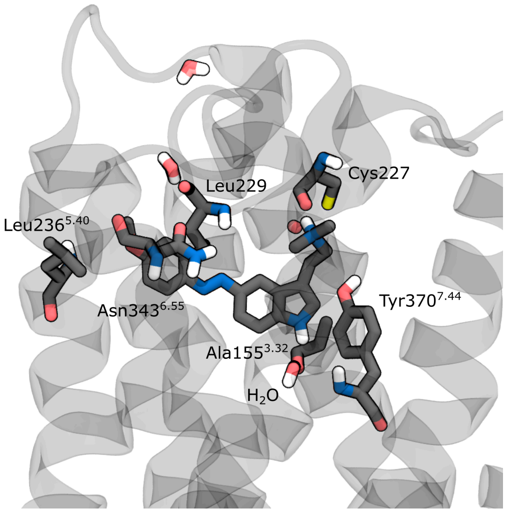

Figure S26: Binding mode of trans-1 in the D155A mutant. Key residues lining the binding pocket are shown as sticks: Asn343$^{6.55}$, Leu236$^{5.40}$, Tyr370$^{7.44}$, and ECL2 residues Leu229 and Cys227. The bridging water molecule is labeled. Receptor backbone shown as grey cartoon.

In the T160A mutant, the impact of the loss of the Thr160$^{3.37}$ contact depends on the receptor state. In the inactive receptor, the distance between the *trans*-**1** methoxy group and Asp231$^{5.35}$ shows a bimodal distribution (Figure S27): the dominant population (70%) maintains hydrogen-bonding distances below 3Å, while a secondary population (~30%) shows distances of 7 Å, indicating the cleavage of this contact. In the active receptor, the Asp231$^{5.35}$ interaction is more consistently preserved. By contrast, the hydrogen bond between the indole NH and Ser242$^{5.46}$ remains stable regardless of receptor state and is maintained for both *trans*-**1** and *cis*-**2** isomers (Figure S28).

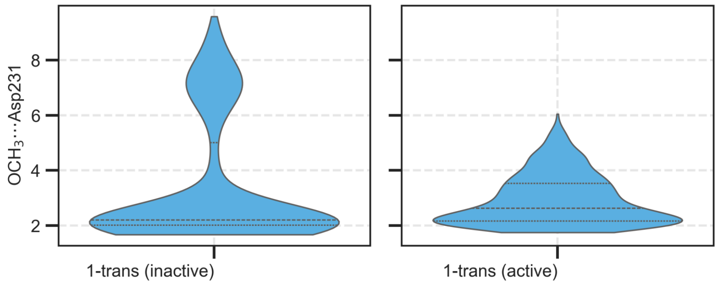

Figure S27: Violin plot of the OCH_3···Asp231$^{5.35}$ distance in T160A.

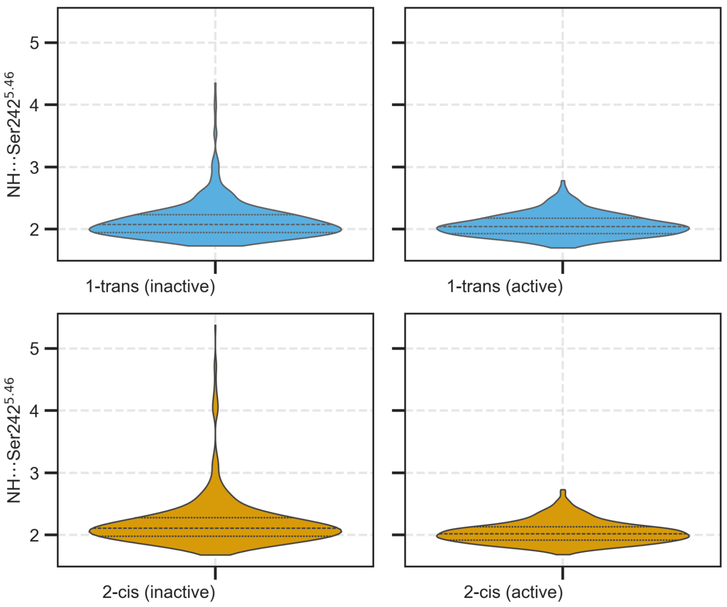

Figure S28: Violin plot of the NH···Ser242$^{5.46}$ distance in T160A for trans-1 (blue) and cis-2 (orange).

For D231P, 1-trans remains anchored within the binding pocket of the inactive receptor via Thr160$^{3.37}$ (Figure S29). On the other side, when bound to the active receptor, the attachment points are provided by either Ser242$^{5.46}$ or Thr160$^{3.37}$ (Figure S29,S30), reproducing the results observed with the wild type.

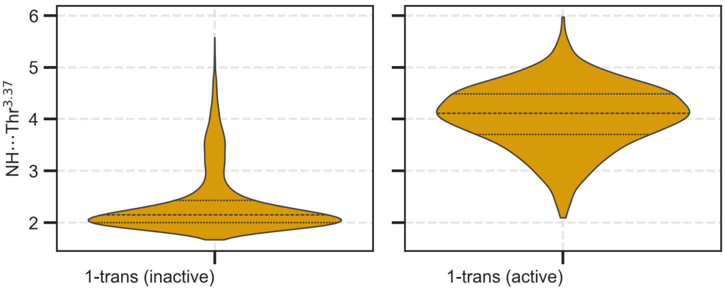

Figure S29: Violin plot of the NH···Thr160$^{3.37}$ distance in D231P

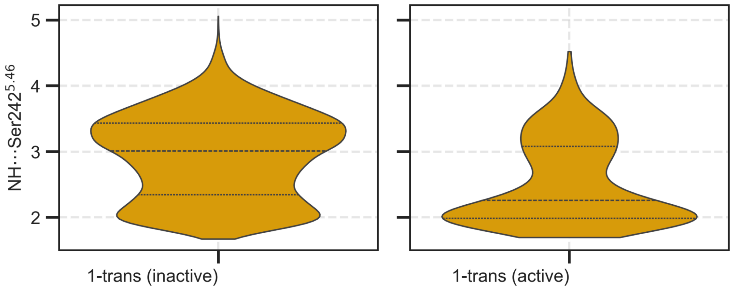

Figure S30: Violin plot of the NH···Ser242$^{5.46}$ distance in D231P.

These results support our central finding that the hydrogen bond network involving Asp231$^{5.35}$, Thr160$^{3.37}$, and Ser242$^{5.46}$ provides redundant anchoring points, and that the pharmacological differences between compounds 1 and 2 arise from their differential ability to engage this network rather than from a single critical interaction.

Supplementary Note 5: Trajectory Analysis

All analyses were performed exclusively on the productive portion of the MD trajectories. The productive trajectories were processed and analyzed using the CPPTRAJ program (version 6.29.13) from the AmberTools suite. For the hydrogen bond analyses, we used a cutoff of 3.2 Å and 125° for the distance and angle, respectively. Ligand insertion depth was quantified by aligning each ligand–receptor complex to the LSD-bound active-state cryo-EM structure (PDB 9AS3) and

computing the RMSD between the nine atoms of the ligand's indole core and the corresponding nine atoms of LSD's indole core. Because the indole moiety anchors the ligand at the base of the orthosteric pocket, this RMSDi provides a direct measure of vertical insertion depth relative to LSD. The same procedure was applied to the inactive-like receptor using its LSD-bound X-ray structure as the reference. The RMSDi was computed at every 0.1 ns according to:

$$RMSD_i = \sqrt{\frac{1}{9}\sum_{i=1}^{9}\left|r_i^{lig} - r_i^{LSD}\right|^2}$$

The movement of the toggle switch is described by the two dihedral angles χ1 (N–Cα–Cβ–Cγ) and χ2 (Cα–Cβ–Cγ–Cδ1). These dihedrals define the orientation of the side chain relative to the backbone and the indole ring. While the rotamer population of χ2 clusters between 90° and 150°, a χ1 rotamer population clustering around −80° is associated with the inactive state receptor while a rotamer population clustering around −160° is defined as a hallmark of the active state receptor. A salt bridge is a noncovalent electrostatic interaction formed between oppositely charged functional groups. Salt bridges are identified when the distance between the charged heteroatoms falls below ~4 Å.

Supplementary References

1. Zhao, S., Wei, H., Cieplak, P., Duan, Y. & Luo, R. PyRESP: A program for electrostatic parameterizations of additive and induced dipole polarizable force fields. *J. Chem. Theory Comput.* **18**, 3654–3670 (2022).
2. Duchstein, P., Neiss, C., Görling, A. & Zahn, D. Molecular mechanics modeling of azobenzene-based photoswitches. *J. Mol. Model.* **18**, 2479–2482 (2012).
3. Kristiansen, K. *et al.* A highly conserved aspartic acid (asp-155) anchors the terminal Amine moiety of tryptamines and is involved in membrane targeting of the 5-HT2A serotonin receptor but does not participate in activation via a 'salt-bridge disruption' mechanism. *J. Pharmacol. Exp. Ther.* **293**, 735–746 (2000).
